# Metabolic dysfunction-associated fatty liver disease and risk of knee osteoarthritis: A prospective cohort study

**DOI:** 10.1007/s10238-026-02096-5

**Published:** 2026-03-05

**Authors:** Yongchun Zhang, Lina Jiang, Xiaowen Teng, Chenxi Xia, Haofeng Zhou, Fang Wang

**Affiliations:** 1 Department of Orthopedics, The Affiliated Qingdao Third People’s Hospital of Qingdao University, Qingdao, China; 2 Department of Cardiology, Qingdao Eighth People’s Hospital, Qingdao, China; 3 Department of Cardiology, Beijing Hospital, National Center of Gerontology, Institute of Geriatric Medicine, Chinese Academy of Medical Sciences, Beijing, China; 4 Graduate School, Peking Union Medical College, Chinese Academy of Medical Science, Beijing, China; 5 Beijing Key Laboratory of Geriatrics-Based and Al-Driven Pharmaceutical and Medical Device Evaluation and Translation, Beijing, China

**Keywords:** Metabolic dysfunction-associated fatty liver disease, Knee osteoarthritis, Systemic inflammation, Cohort study

## Abstract

Knee osteoarthritis (KOA) is a leading cause of chronic pain and disability. Metabolic dysfunction-associated fatty liver disease (MAFLD) is a systemic metabolic disorder that influences extra-hepatic conditions. Although cross-sectional studies link MAFLD to KOA, prospective evidence remains limited. This study aimed to investigate the longitudinal association between MAFLD, and KOA and assess the mediating role of inflammation. This study included 303,604 participants from the UK Biobank without baseline osteoarthritis. MAFLD was defined using the fatty liver index alongside metabolic criteria, fibrosis severity was assessed using the Fibrosis-4 score, and MAFLD subtypes were categorized. Incident KOA was identified through linked health records. Cox proportional hazard regression model was used to estimate hazard ratios (HR) and 95% confidence interval (CI) for the association. Mediation analysis evaluated the potential role of high-sensitivity C-reactive protein (hs-CRP). Over a median follow-up of 13.67 years, 17,137 KOA cases occurred. MAFLD was associated with an 18% higher risk of KOA (HR 1.18, 95% CI 1.13–1.24), with risk increasing by fibrosis severity (P for trend < 0.001). Among subtypes, MAFLD-overweight/obesity showed a significant association with KOA (HR 1.19, 95% CI 1.14–1.25), while MAFLD-diabetes (HR 1.05, 95% CI 0.96–1.16) and MAFLD-lean (HR 1.23, 95% CI 0.93–1.62) did not reach statistical significance. Additionally, hs-CRP explained 8.94% of the association between MAFLD and KOA. MAFLD was independently associated with higher KOA risk; inflammation partially mediates this association. These findings suggest MAFLD as a systemic metabolic condition affecting musculoskeletal health, supporting integrated management strategies.

**Supplementary Information:**

The online version contains supplementary material available at 10.1007/s10238-026-02096-5.

## Introduction

Knee osteoarthritis (KOA) is a leading global cause of chronic pain and disability [1]. According to the Global Burden of Disease Study, approximately 370 million individuals are affected by KOA worldwide [2]. This number is projected to reach an estimated 642 million by 2050 due to population aging and the rising prevalence of obesity, imposing a severe health and economic burden [3, 4]. Consequently, identifying modifiable risk factors is crucial for preventive strategies to mitigate the disease burden [5]. 

The etiology of osteoarthritis remains incompletely understood. Current evidence indicates that osteoarthritis is a multifaceted disease influenced by various pathogenic factors [6, 7]. Beyond the well-established roles of ageing and excess body weight, systemic metabolic dysfunction, such as insulin resistance and dyslipidemia, has emerged as a significant contributor to disease pathogenesis [8–10]. Metabolic dysfunction-associated fatty liver disease (MAFLD) is a prototypical manifestation of this systemic metabolic dysregulation [11]. Defined by hepatic steatosis concurrent with metabolic abnormalities, MAFLD is not merely a liver-specific disorder but a marker of pervasive metabolic disturbance [12]. Evidence has linked MAFLD to an elevated risk of various extra-hepatic diseases, including cardiovascular disease, renal failure and neurological disorders [13–15]. Cross-sectional studies have described an association between MAFLD and KOA. For example, a Korean national study reported individuals with MAFLD had a 1.48-fold higher risk of KOA [16]. However, the cross-sectional design of existing studies precludes the establishment of temporal sequence and causality. Therefore, whether MAFLD is independently associated with incident KOA in longitudinal settings remains unclear. Furthermore, the MAFLD definition classifies patients into three homogenous subtypes, and different subtypes may have distinct causes, progression, and outcomes [17]. Yet, whether these subtypes differentially affect the risk of KOA remains to be determined. Additionally, systemic inflammation is a key pathophysiological feature shared by MAFLD and KOA, while the extent to which it mediates the association has not been evaluated.

To address these gaps, we conducted a prospective cohort study using data from 303,604 participants in the UK Biobank. The aims of this study were to investigate the longitudinal association between MAFLD and the risk of KOA, examine whether this association differs across MAFLD subtypes, and assess the potential mediating role of inflammation, as measured by high-sensitivity C-reactive protein (hs-CRP), in this association.

## Patients and methods

### Study design and participants

This prospective cohort study utilized data from the UK Biobank, a population-based study that recruited over 500,000 participants aged 37–73 years between 2006 and 2010 from 22 assessment centers across the United Kingdom. The study design and methodology have been detailed in prior publications [18]. At baseline, participants completed touch-screen questionnaires, underwent anthropometric measurements, provided biological samples, and participated in imaging and genetic profiling. Follow-up for health outcomes is ongoing through linkage to national health records. The UK Biobank study was approved by the North West Multi-Centre Research Ethics Committee (reference number 11/NW/0382), and all participants provided written informed consent.

**Fig. 1:**
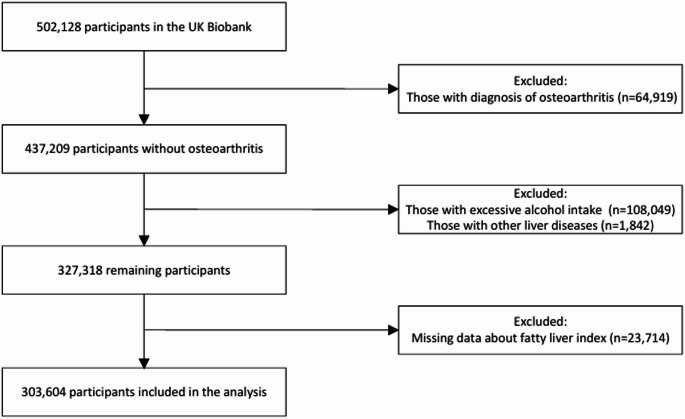
Study flow chart

For the present analysis, we excluded individuals with pre-existing osteoarthritis at baseline (*n* = 64,919), those with excessive alcohol consumption (≥ 30 g/day for men; ≥20 g/day for women, *n* = 108,049), or other chronic liver diseases (*n* = 1,842). We further excluded 23,714 participants with missing data on the fatty liver index (FLI). Finally, a total of 303,604 participants were included in the analysis (Fig. 1).

### Definition of MAFLD

The diagnosis of MAFLD was based on the Asian Pacific association for the study of the liver clinical practice guidelines [19]. MAFLD was defined as the presence of hepatic steatosis in the absence of other chronic liver diseases or excessive alcohol consumption, plus at least one of the following criteria: type 2 diabetes (T2D); overweight or obesity (body mass index (BMI) ≥ 25 kg/m^2^ in Caucasian or ≥ 23 kg/m^2^ in Asian); or metabolic abnormality including any of the two: elevated waist circumference (≥ 102/88 cm for men and women in Caucasian or ≥ 90/80 cm in Asian), hypertension (blood pressure ≥ 130/85 mmHg or use of antihypertensive medication), hypertriglyceridemia (≥ 1.7 mmol/L), low high-density lipoprotein cholesterol (HDL-C) (< 1.0 mmol/L for males; < 1.3 mmol/L for females), prediabetes (fasting glucose 5.6 to 6.9 mmol/L or glycated hemoglobin 5.7% to 6.4%), insulin resistance, and subclinical inflammation (hs-CRP > 2 mg/L). Given the lack of serum insulin data in the UK Biobank, insulin resistance was not considered.

As imaging data were not broadly available in the UK Biobank, hepatic steatosis was assessed using the FLI, calculated from waist circumference, gamma-glutamyl transferase, triglyceride, and BMI [20]. FLI ≥ 60 was used to diagnose hepatic steatosis, which achieve a sensitivity of 87% and specificity of 86% in the original validation, and demonstrate a sensitivity of 81% and specificity of 90% in a meta-analysis of multi-ethnic populations [20, 21]. Given its non-invasive nature and cost-effectiveness, FLI is considered suitable for epidemiological studies to detect hepatic steatosis by current clinical practice guidelines [19, 22]. 

Among participants with MAFLD, disease severity was assessed using the fibrosis 4 (FIB-4) score, calculated as: FIB-4: (age × AST)/(platelets × ALT^0.5^). For advanced liver fibrosis, the lower cutoff is 1.30, and the upper cutoff was 2.67 [23]. Therefore, MAFLD participants were categorized into three groups: early fibrosis (FIB-4 < 1.30), intermediate fibrosis (1.30 ≤ FIB-4 < 2.67), and possible advanced fibrosis (FIB-4 ≥ 2.67). Given the considerable heterogeneity of MAFLD, participants with MAFLD were classified into three subtypes: MAFLD-diabetes (those with a history of diabetes), MAFLD-overweight/obesity (overweight/obese individuals without diabetes), and MAFLD-lean (lean individuals without diabetes but with ≥ 2 metabolic abnormalities) [17]. 

### Assessment of inflammatory mediator

Systemic inflammation was assessed using baseline hs-CRP levels. Blood samples were collected from consenting participants, separated by com ponents and stored at UK Biobank (−80˚C and LN_2_) until analysis. Blood biomarkers were conducted externally with stringent quality control; assay performance details are given elsewhere [24]. 

### Ascertainment of outcome

Incident KOA was ascertained from linked hospital inpatient records, primary-care files, and self-reported medical histories. The 9th and 10th revisions of the International Classification of Diseases and self-reported data fields with choice-, disease-, or procedure-specific codes were listed in Table S1, which has a positive predictive value of 88% for KOA [25, 26]. Follow-up for each participant started at the baseline assessment date and ended at the date of the first KOA diagnosis, date of death, or the end of the available follow-up, whichever occurred first. At the time of this analysis, the latest available follow-up data were until October 31, 2022, for England; August 31, 2022, for Scotland; and May 31, 2022, for Wales.

### Covariates

The following baseline variables were treated as potential confounders: age (years, continuous), sex, ethnicity (White or others), educational attainment (college/university degree or other), Townsend deprivation index (continuous), physical activity (MET-minutes/week, continuous), smoking status (never, former or current), daily ethanol intake (g/day, continuous), diet quality score (0, 1, 2 and 3 point), insomnia (never/rarely, sometimes or usually), BMI (kg/m^2^, continuous), and laboratory measures, including HDL-C, low-density lipoprotein cholesterol (LDL-C), total cholesterol, and triglyceride. Self-reported medical history variables included T2D, hypertension, and any prior fracture. Age, sex, ethnicity, education, smoking, alcohol, diet, insomnia, and medical history were collected from touchscreen questionnaire; blood lipids were obtained from fasting serum assays. Diet quality was assessed based on intake of fruit and vegetables, fish, processed and red meat, where high scores indicated healthy dietary [27]. Missing values were multiply-imputed using chained-equation regression.

### Statistical analysis

Baseline characteristics were presented by MAFLD status. Continuous variables with normal distribution were expressed as mean (standard deviation, SD) and compared using Student’s *t* test; non-normally distributed variables were expressed as median (interquartile range, IQR) and were compared with the Mann-Whitney U test. Categorical variables were presented as frequencies (percentages), and compared using the chi-square test.

Cox proportional hazards regression was used to estimate hazard ratios (HRs) and 95% CIs for the association between MAFLD and incident KOA. The proportional hazards assumption was assessed using the Schoenfeld residuals method. Three models were constructed: Model 1 was unadjusted. Model 2 adjusted for age, sex, ethnicity, education level and Townsend deprivation index. Model 3 additionally adjusted for physical activity, smoking status, daily ethanol intake, diet quality score, insomnia, BMI, HDL-C, LDL-C, total cholesterol, triglyceride, and history of T2D, hypertension, and fracture. The associations of fibrosis severity and MAFLD subtypes with KOA were also evaluated using Cox proportional hazards regression models, with non-MAFLD group as the reference. Kaplan-Meier survival curves were plotted to compare the cumulative incidence of KOA between the non-MAFLD group and MAFLD participants stratified by fibrosis severity, with the log-rank test used to evaluate differences.

We employed a causal mediation analysis framework using the CMAverse R package to estimate the proportion of the association mediated by hs-CRP. Models were adjusted for all covariates in Model 3. We calculated the indirect effect, direct effect, and total effect. The mediation proportion was calculated as indirect effect divided by total effect, and 95% CI were derived from 1000 non-parametric bootstraps.

Subgroup analyses by age (< 60, ≥ 60 years), sex (female, male), BMI (< 25, 25–29.9.9, ≥ 30 kg/m^2^), smoking status (never, former/current), physical activity (< 600 MET- min/week, ≥ 600–1200 MET- min/week). Interaction was tested by including a multiplicative term in the fully adjusted Cox model. To test the robustness of our findings, we conducted three sensitivity analyses. First, we excluded participants who developed KOA within the first 2 year to minimize the potential reverse causation. Second, we constructed Fine and Gray proportional subdistribution hazards regression models to account for the possible competing risk of death. Third, we performed a complete-case analysis by excluding all participants with missing values for any covariates included in model 3.

A two-sided P-value < 0.05 was considered statistically significant. All analyses were performed using R software (version 4.2.1).

## Results

### Baseline characteristics of the participants

The study included 303,604 participants with a mean age of 56.0 years, of whom 44.6% were male. 106,565 (35.1%) met the criteria for MAFLD. Baseline characteristics of the overall cohort and stratified by MAFLD status are presented in Table 1. Compared with participants without MAFLD, those with MAFLD were more likely to be older, male, White, and had a lower educational attainment and socioeconomic status. The MAFLD group also had a higher mean BMI, were more likely to be current smokers, reported higher alcohol intake and lower physical activity levels, and had a less favorable dietary pattern. Clinically, this group showed a more adverse lipid profile, significantly elevated hs-CRP level and a higher prevalence of T2D and hypertension.

**Table 1:** Baseline characteristics of participants

Characteristic	Total (*n* = 303604)	Non-MAFLD (*n* = 197039)	MAFLD (*n* = 106565)	*P*
Age, years	55.97(8.22)	55.59(8.32)	56.66(7.99)	< 0.001
Male	135,486(44.6)	69,401(35.2)	66,085(62.0)	< 0.001
White ethnicity	281,478(92.7)	183,114(92.9)	98,364(92.3)	< 0.001
College or university degree	99,117(33.1)	70,489(36.2)	28,628(27.3)	< 0.001
Townsend Deprivation Index	−2.16[−3.66,0.54]	−2.29[−3.72,0.25]	−1.89[−3.50,1.05]	< 0.001
Smoking status				< 0.001
Never	183,249(60.4)	126,040(64.0)	57,209(53.7)	
Former	92,340(30.4)	54,064(27.4)	38,276(35.9)	
Current	28,015(9.2)	16,935(8.6)	11,080(10.4)	
Alcohol intake, g/day	6.76[0.00,13.53]	6.76[0.00,13.53]	6.76[0.00,15.78]	< 0.001
Diet score				< 0.001
0	47,821(15.8)	27,202(13.8)	20,619(19.3)	
1	113,923(37.5)	72,248(36.7)	41,675(39.1)	
2	101,305(33.4)	68,483(34.8)	32,822(30.8)	
3	40,555(13.4)	29,106(14.8)	11,449(10.7)	
Physical activity, MET-min/week	1782.00 [815.00, 3559.50]	1938.00[927.50,3773.75]	1512.00[655.00,3159.00]	< 0.001
Insomnia				< 0.001
Never/rarely	78,043(25.7)	50,985(25.9)	27,058(25.4)	
Sometimes	146,122(48.1)	96,357(48.9)	49,765(46.7)	
Usually	79,439(26.2)	49,697(25.2)	29,742(27.9)	
BMI, kg/m2	27.21(4.76)	24.85(2.87)	31.61(4.46)	< 0.001
hs-CRP	2.52(4.25)	1.91(3.77)	3.63(4.83)	< 0.001
Triglycerides, mmol/L	1.72(1.00)	1.36(0.64)	2.40(1.19)	< 0.001
Total cholesterol, mmol	5.65(1.14)	5.64(1.10)	5.66(1.22)	< 0.001
HDL-C, mmol	1.41(0.36)	1.52(0.36)	1.21(0.28)	< 0.001
LDL-C, mmol	3.55(0.87)	3.51(0.84)	3.62(0.92)	< 0.001
Hypertension	74,732(24.6)	34,709(17.6)	40,023(37.6)	< 0.001
Type 2 diabetes	8003(2.6)	2177(1.1)	5826(5.5)	< 0.001
History of Fracture	26,777(8.8)	17,397(8.8)	9380(8.8)	0.739

MAFLD, metabolic dysfunction-associated fatty liver disease; hs-CRP, high-sensitivity C-reactive protein; HDL-C, high-density lipoprotein cholesterol; LDL-C, low-density lipoprotein cholesterol

### Association between MAFLD and risk of KOA

During a median follow-up period of 13.67 (IQR 12.95–14.39) years, 9,044 cases of KOA were recorded among participants with MAFLD, and 8,093 among those without MAFLD. In the Cox regression analysis, MAFLD was associated with an 18% higher risk of developing KOA after adjustment for potential confounders (HR 1.18, 95% CI 1.13–1.24) (Table 2). The baseline characteristics of the MAFLD subjects with different fibrosis severity were shown in Table S2. Compared with the non-MAFLD group, the adjusted HRs for incident KOA across increasing severity categories were 1.17 (95% CI 1.12–1.23) for the early fibrosis group, 1.19 (95% CI 1.13–1.25) for the intermediate fibrosis group, and 1.21 (95% CI 1.05–1.38) for the possible advanced fibrosis group (P for trend < 0.001). The Kaplan-Meier curves for cumulative KOA incidence across these groups are presented in Figure S1.

According to the classification criteria, 5,826 MAFLD participants were in the MAFLD-diabetes group, 99,270 in the MAFLD-overweight/obese group, and 1,469 in the MAFLD-lean group. The baseline characteristics of these subtypes are presented in Table S3. After full adjustment, the association with incident KOA varied across MAFLD subtypes (Table 2). Compared with the non-MAFLD group, the MAFLD-overweight/obesity subtype was associated with a 19% higher risk of KOA (HR 1.19, 95% CI 1.14–1.25). However, the MAFLD-diabetes subtype (HR 1.05, 95% CI 0.96–1.16; *P* = 0.244) and the MAFLD-lean subtype (HR 1.23, 95% CI 0.93–1.62; *P* = 0.138) showed associations with KOA that did not reach statistical significance.

**Table 2:** Associations of MAFLD with incident knee osteoarthritis

	Events/person-years	Model 1		Model 2		Model 3	
		HR (95%CI)	*P*	HR (95%CI)	*P*	HR (95%CI)	*P*
Non-MAFLD	8093/2,654,037	Reference		Reference		Reference	
MAFLD	9044/1,403,669	2.11 (2.05–2.18)	< 0.001	2.08 (2.01–2.14)	< 0.001	1.18 (1.13–1.24)	< 0.001
**Fibrosis severity**							
FIB-4 < 1.30	4970/861,347	1.89 (1.83–1.97)	< 0.001	2.10 (2.02–2.19)	< 0.001	1.17 (1.12–1.23)	< 0.001
1.30 ≤ FIB-4 < 2.67	3840/513,860	2.45 (2.36–2.55)	< 0.001	2.13 (2.05–2.21)	< 0.001	1.19 (1.13–1.25)	< 0.001
FIB-4 ≥ 2.67	234/28,462	2.70 (2.37–3.07)	< 0.001	2.20 (1.93–2.51)	< 0.001	1.21 (1.05–1.38)	0.007
P for trend			< 0.001		< 0.001		< 0.001
**MAFLD subtypes**							
MAFLD-diabetes	565/76,055	2.43 (2.24–2.66)	< 0.001	2.11 (1.94–2.30)	< 0.001	1.05 (0.96–1.16)	0.244
MAFLD-overweight/obese	8427/1,307,679	2.11 (2.05–2.18)	< 0.001	2.13 (2.07–2.21)	< 0.001	1.19 (1.14–1.25)	< 0.001
MAFLD-lean	52/19,935	0.86 (0.65–1.12)	0.262	0.84 (0.64–1.11)	0.221	1.23 (0.93–1.62)	0.138

MAFLD, metabolic dysfunction-associated fatty liver disease; FIB-4, Fibrosis-4

Model 1 was unadjusted. Model 2 adjusted for age, sex, ethnicity, education level and Townsend deprivation index. Model 3 additionally adjusted for physical activity, smoking status, daily ethanol intake, diet quality score, insomnia, body mass index, high-density lipoprotein cholesterol, low-density lipoprotein cholesterol, total cholesterol, triglyceride, and history of type 2 diabetes, hypertension, and fracture

### Mediation analysis

**Fig. 2:**
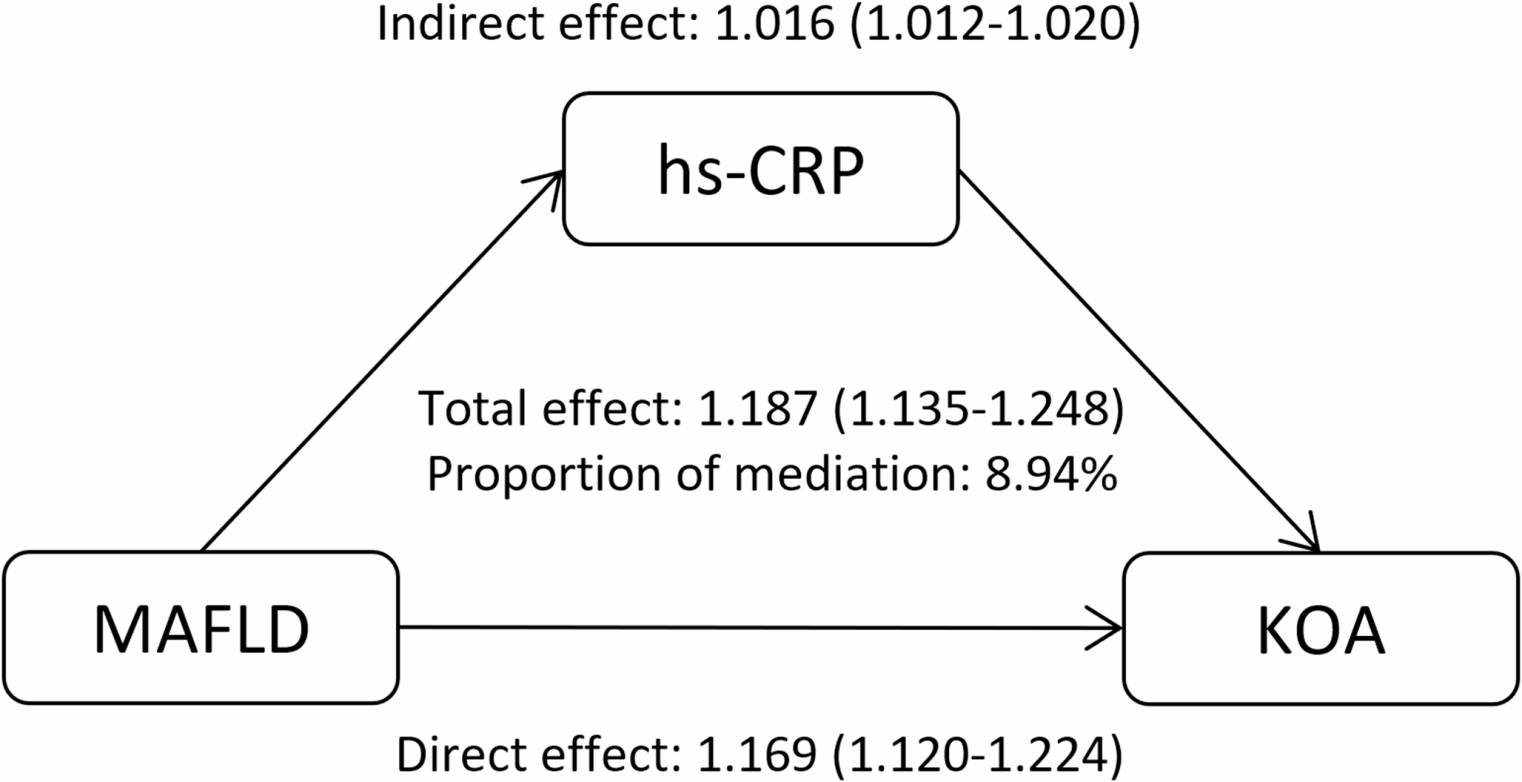
Estimated proportion of the association between metabolic dysfunction-associated fatty liver disease and knee osteoarthritis mediated by high-sensitivity C-reactive protein

Mediation analysis revealed that hs-CRP significantly mediated the association between MAFLD and knee osteoarthritis, as shown in Fig. 2. After full adjustment for sociodemographic characteristics, lifestyle factors, lipid markers, and comorbidities, the total effect of MAFLD on KOA risk was 1.187 (95% CI 1.135–1.248). This effect decomposed into a direct effect of 1.169 (95% CI 1.120–1.224) and an indirect effect mediated through hs-CRP of 1.016 (95% CI 1.012–1.020). The proportion of the total effect mediated by hs-CRP was 8.94%.

### Subgroup and sensitivity analyses

**Fig. 3:**
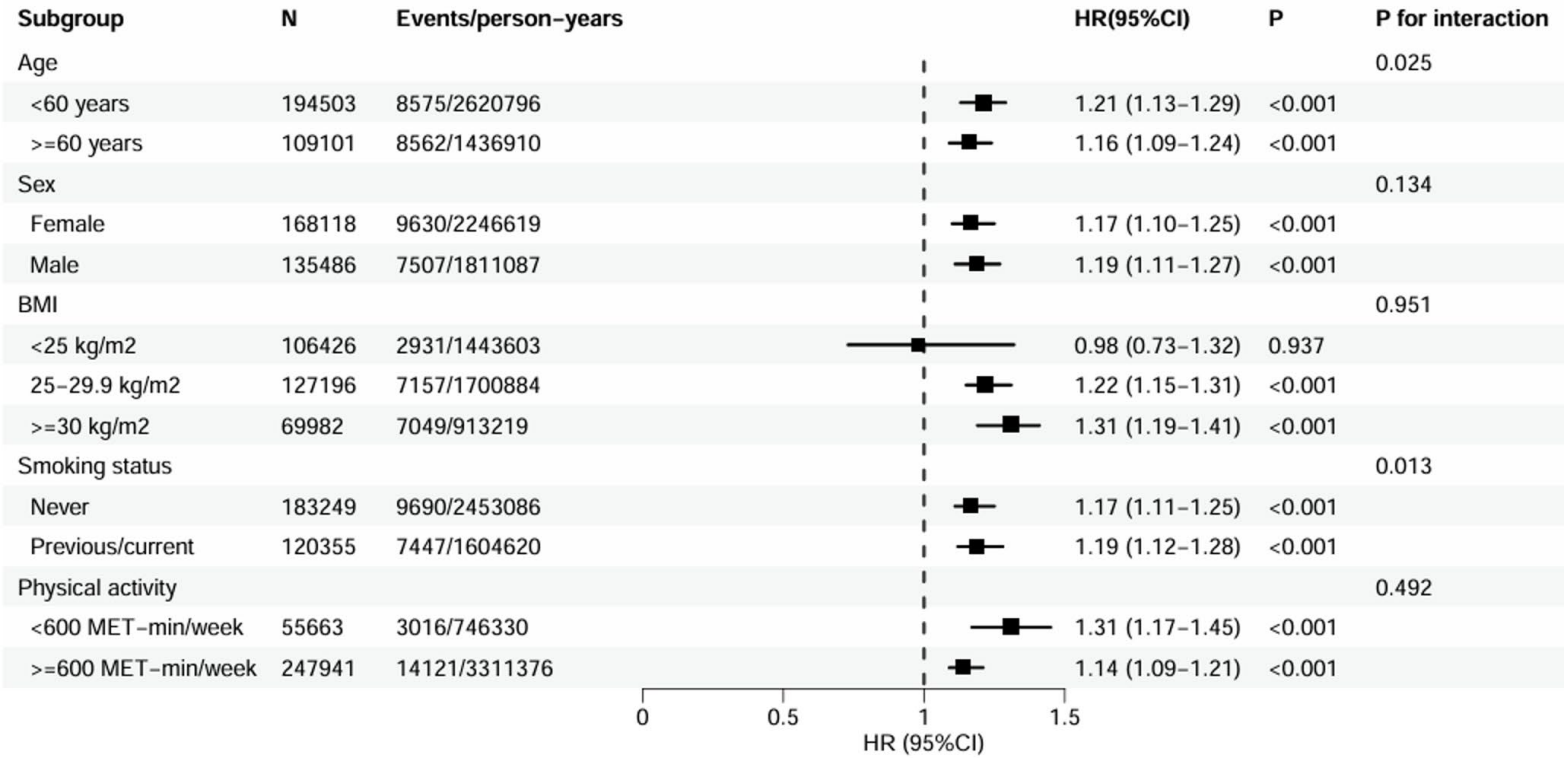
Subgroup analyses for the association of metabolic dysfunction-associated fatty liver disease with knee osteoarthritis

Subgroup analyses are summarized in Fig. 3. Significant effect modification was observed for age and smoking status (P for interaction = 0.025 and 0.013, respectively). The association appeared slightly stronger among participants aged < 60 years (HR 1.21, 95% CI 1.13–1.29) compared with those aged ≥ 60 years (HR 1.16, 95% CI 1.09–1.24). A marginal difference was also noted between former/current smokers (HR 1.19, 95% CI 1.12–1.28) and never smokers (HR 1.17, 95% CI 1.11–1.25). No significant interaction was detected for sex, BMI, or physical activity level (all P for interaction > 0.05). In the sensitivity analyses, the primary findings remained generally robust when excluding participants who developed KOA within the first two years of follow-up, using competing risk models accounting for death, or excluding individuals with missing covariate data (Table S4).

## Discussion

In this large-scale prospective cohort study of over 300,000 participants from the UK Biobank, we found that MAFLD was independently associated with an 18% higher risk of incident KOA, and this risk exhibited a dose-response relationship with the severity of MAFLD, as assessed by the FIB-4 score. The association between MAFLD and KOA varied substantially across subtypes, with the overweight/obesity subtype showing a 19% higher risk, whereas the diabetes and lean subtypes did not reach statistical significance. Furthermore, mediation analysis showed that hs-CRP explained 8.94% of the association. These findings advance our understanding of MAFLD as a systemic metabolic disorder with significant extra-hepatic consequence.

Our findings are consistent with the association between MAFLD and an increased risk of KOA reported in cross-sectional studies and extend this evidence. In a Korean national survey, Han et al. reported that MAFLD was associated with a 1.48-fold higher probability of radiographic KOA among adults aged ≥ 50 years [16]. Similarly, analyses of the US National Health and Nutrition Examination Survey (NHANES) data found that NAFLD was associated with a 1.72-fold increased risk of self-reported OA after multivariable adjustment (odds ratio 1.72, 95% CI 1.26–2.34) [28]. Another NHANES-based study further observed a positive correlation between hepatic steatosis, assessed by controlled attenuation parameter, and osteoarthritis [29]. Collectively, have suggested a consistent link between fatty liver disease and OA across diverse populations and diagnostic criteria. This prospective analysis extends prior evidence by establishing a temporal sequence between MAFLD and KOA, supporting the hypothesis that MAFLD is associated with a higher risk of KOA.

A key contribution of our work is the identification of a severity-dependent risk gradient. We found that the risk of KOA incrementally increased across categories of MAFLD severity defined by FIB-4 scores. A prior cross-sectional analysis of US adults reported no significant association between liver fibrosis and arthritis (including osteoarthritis) [29]. However, the cross-sectional design limited the ability to infer temporality or causality, and its sample size may have constrained statistical power to detect modest associations. This prospective cohort study of large sample size, with long-term follow-up provide more robust power to detect a significant gradient in risk related to MAFLD severity. This finding extends beyond the binary presence or absence of MAFLD and suggests that the pathophysiological burden of the liver disease, particularly the progression towards fibrosis, may amplify its impact on joint health. This concept of severity-dependent risk is consistent with findings in other MAFLD-related extra-hepatic complications, including cardiovascular disease, chronic kidney disease, and dementia [15, 30, 31]. 

This study also investigated the impact of MAFLD subtypes on the risk of KOA. The overweight/obesity subtype, which constituted the vast majority of MAFLD cases, showed a risk of KOA comparable to that of the overall MAFLD cohort. This suggests that the overweight/obesity subtype largely reflects the KOA risk association in the overall MAFLD population. For the diabetes subtype, no significant association was found in the primary Cox model. However, when accounting for death as a competing risk, an association of borderline significance emerged, implying that the competing risk of mortality in this older, higher-risk population may have masked the association with incident KOA. Regarding the lean MAFLD subtype, the point estimate also indicated an elevated risk of KOA; however, this association was not statistically significant, likely due to the limited sample size of this subgroup. This trend suggests that metabolic dysfunction may contribute to joint pathology even in the absence of obesity, a hypothesis requires validation in larger cohorts.

The observed association between MAFLD and KOA might stem from several pathways. MAFLD is associated with chronic low-grade inflammation, with hepatic steatosis driving the release of pro-inflammatory cytokines (e.g., IL-6, TNF-α) and elevating systemic markers [32, 33]. Our mediation analysis revealed that hs-CRP mediated 8.94% of the association between MAFLD and risk of KOA, consistent with prior evidence on metabolic syndrome and osteoarthritis [34]. Circulating mediators promote a pro-inflammatory joint microenvironment, activate synovial cells and accelerate cartilage catabolism [35]. Furthermore, MAFLD is intrinsically linked to dyslipidemia, which can impair joint homeostasis via ectopic lipid deposition in chondrocytes, and through the action of oxidized low-density lipoprotein to stimulate cartilage-degrading enzymes [36, 37]. MAFLD also alters adipokine secretion, notably increased leptin, which can directly promote inflammation and inhibit cartilage repair within the joint [38]. Additionally, oxidative stress, another characteristic of MAFLD pathogenesis, may further exacerbate joint damage through chondrocyte apoptosis and extracellular matrix degradation [32, 39]. The observed dose-response relationship with fibrosis severity suggests that the intensity of these systemic pathological signals may be amplified with advancing liver disease. A recent preclinical study found that mice with advanced fibrosis exhibited greater subchondral bone loss, cartilage erosion, and joint inflammation compared to those with simple steatosis independent of severe obesity [40]. 

Subgroup analyses found that the association between MAFLD and KOA was slightly stronger among participants aged < 60 years, which may reflect a more pronounced impact of metabolic dysfunction and inflammation on joint homeostasis in earlier adulthood, or differential survival and competing risks in older populations. Besides, a significant interaction was also observed with smoking status, with former or current smokers exhibiting a marginally stronger association than never smokers. This could reflect the effects of smoking on systemic inflammation and oxidative stress, pathways implicated in both MAFLD and OA pathogenesis [41]. However, the magnitude of these differences was modest. These subgroup findings should therefore be considered exploratory, and their clinical relevance requires confirmation in future studies.

This study has several clinical implications. It highlights the need for clinicians managing MAFLD to be aware of the increased risk of KOA, potentially facilitating earlier symptom recognition and multidisciplinary care. More importantly, the shared metabolic and inflammatory pathways suggest the potential for integrated management. For instance, lifestyle interventions targeting weight loss and improved metabolic health, which are first-line for MAFLD, may concurrently delay or mitigate the progression of KOA by reducing systemic inflammation and metabolic stress [42]. 

Our study also has notable strengths, including its prospective design, large sample size, long follow-up duration, and comprehensive phenotypic. Besides, the use of several sensitivity analyses enhanced the robustness of our findings. However, there are several limitations in this study. First, MAFLD was defined using the FLI rather than imaging or histology, which may introduce misclassification. Prior validation studies demonstrated that FLI exhibits reduced diagnostic accuracy in lean individuals and African-ancestry populations [43, 44]. Second, OA events were identified solely based on hospital admission records and death registers, without standardized imaging to assess baseline KOA severity or track structural progression. This may result in outcome misclassification and precludes examination of whether the association between MAFLD and KOA differs by baseline severity. Third, the UK Biobank cohort is predominantly of European ancestry and may limit the applicability of our findings to other ethnic groups. Future studies validating these associations in multi-ethnic populations are warranted to ensure broader generalizability. Fourth, despite extensive adjustment, residual confounding from unmeasured factors such as genetic background, bone mineral density, and detailed occupational biomechanical loading cannot be excluded. Finally, hs-CRP was measured only at baseline, and changes over time were not captured.

## Conclusion

In conclusion, this large-scale prospective cohort study found that MAFLD was independently associated with a higher risk of KOA. This risk increased with the severity of hepatic fibrosis and varied across MAFLD subtypes. Furthermore, systemic inflammation partially mediated the association between MAFLD and KOA. These findings underscore the necessity for integrated clinical approaches that address the shared inflammatory and metabolic basis of both conditions, potentially informing preventive strategies and overall patient management.

## Supplementary Information

Below is the link to the electronic supplementary material.

Supplementary Material 1

## Data Availability

The data of this study are available from UK Biobank (https://www.ukbiobank.ac.uk/), but restrictions apply to the availability of these data, which were used under license for the current study, and so are not publicly available. Data are however available from the authors upon reasonable request and with permission of UK Biobank.
