# Supplementary material for: Metabolic dysfunction-associated fatty liver disease and risk of knee osteoarthritis: A prospective cohort study
Source: Clin Exp Med. 2026 Mar 5;26(1):182. doi: 10.1007/s10238-026-02096-5 (PMC12979261; doi:10.1007/s10238-026-02096-5)
Supplement: Supplementary file 1 — Supplementary Material 1 [file 10238_2026_2096_MOESM1_ESM.docx]

Figure S1. Kaplan-Meier curves for cumulative KOA incidence across participants without MAFLD and MAFLD participants across different fibrosis severity. MAFLD, metabolic dysfunction-associated fatty liver disease; FIB-4, Fibrosis-4.


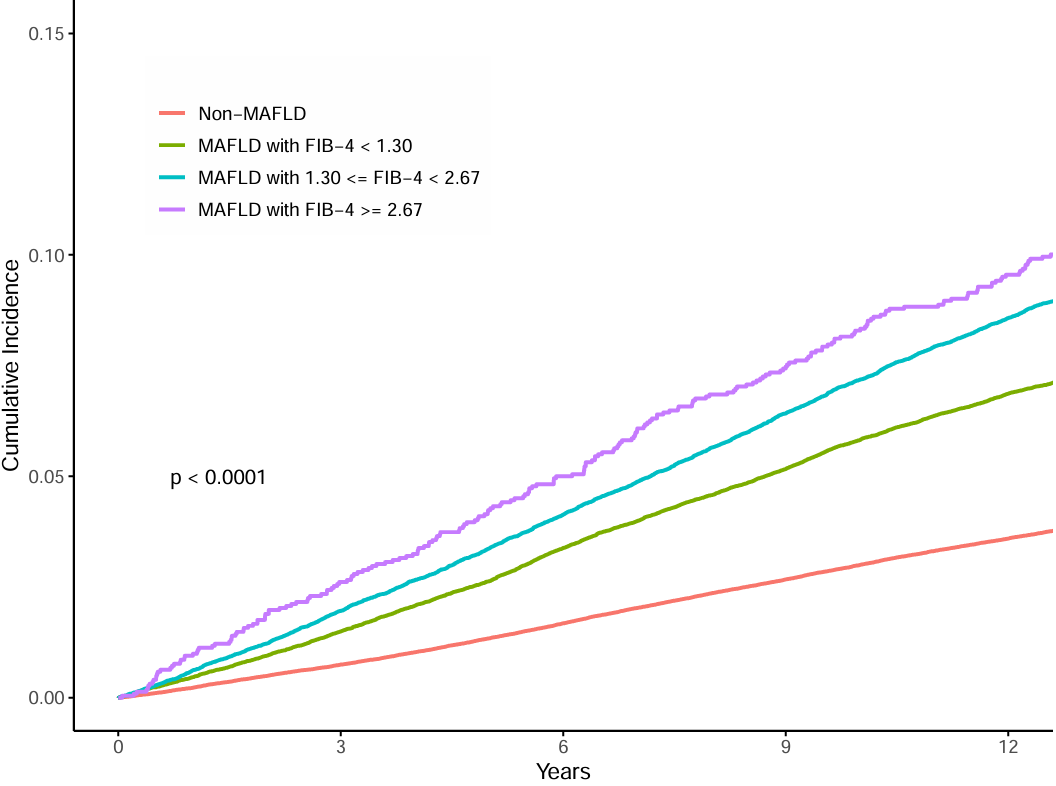


Table S1. Codes used to identify cases of knee osteoarthritis

|  | Field | Code |
| --- | --- | --- |
| ICD-9 | 41271 | 71516, 71536 |
| ICD-10 | 41270 | M17 |
| OPSC4 | 41272 | W40.1, W41.1, W42.1, O18.1 |
| Self-reported | 20002 | 1465 |

ICD-9, International Classification of Diseases, the Ninth Revision; ICD-10, International Classification of Diseases, the Tenth Revision; OPSC4, Office of Population Censuses and Surveys Classification of Interventions and Procedures, version 4.

Table S2. Baseline characteristics of MAFLD participants with different fibrosis severity

| Characteristic | FIB-4<1.30 (n=64829) | 1.30≤FIB-4<2.67 (n=39516) | FIB-4≥2.67 (n=2220) |
| --- | --- | --- | --- |
| Age, years | 53.89(7.76) | 60.88(6.26) | 62.47(6.02) |
| Male | 36801(56.8) | 27607(69.9) | 1677(75.5) |
| White ethnicity | 59499(91.8) | 36827(93.2) | 2038(91.8) |
| College or university degree | 18236(28.6) | 9863(25.4) | 529(24.4) |
| Townsend Deprivation Index | -1.78[-3.45,1.22] | -2.05[-3.58,0.75] | -1.94[-3.55,1.24] |
| Smoking status |  |  |  |
| Never | 36127(55.7) | 20003(50.6) | 1079(48.6) |
| Former | 21033(32.4) | 16292(41.2) | 951(42.8) |
| Current | 7669(11.8) | 3221(8.2) | 190(8.6) |
| Alcohol intake, g/day | 4.72[0.00,14.65] | 6.76[0.00,15.78] | 7.42[0.00,16.29] |
| Diet score |  |  |  |
| 0 | 13317(20.5) | 6928(17.5) | 374(16.8) |
| 1 | 25688(39.6) | 15177(38.4) | 810(36.5) |
| 2 | 19326(29.8) | 12709(32.2) | 787(35.5) |
| 3 | 6498(10.0) | 4702(11.9) | 249(11.2) |
| Physical activity,  MET-min/week | 1434.00[607.50,3030.00] | 1626.00[700.00,3348.00] | 1746.00[744.00,3552.00] |
| Insomnia |  |  |  |
| Never/rarely | 16446(25.4) | 10053(25.4) | 559(25.2) |
| Sometimes | 30171(46.5) | 18583(47.0) | 1011(45.5) |
| Usually | 18212(28.1) | 10880(27.5) | 650(29.3) |
| BMI, kg/m2 | 31.88(4.63) | 31.17(4.13) | 31.31(4.35) |
| C-reactive protein | 3.88(5.08) | 3.20(4.36) | 3.46(4.54) |
| Triglycerides, mmol/L | 2.43(1.20) | 2.35(1.16) | 2.24(1.25) |
| Total cholesterol, mmol | 5.78(1.19) | 5.49(1.24) | 5.09(1.25) |
| HDL-C, mmol | 1.21(0.27) | 1.21(0.28) | 1.18(0.33) |
| LDL-C, mmol | 3.71(0.90) | 3.49(0.93) | 3.19(0.92) |
| Hypertension | 21791(33.7) | 17088(43.4) | 1118(50.6) |
| Type 2 diabetes | 3249(5.0) | 2360(6.0) | 217(9.8) |
| History of Fracture | 5906(9.1) | 3287(8.3) | 187(8.4) |

MAFLD, metabolic dysfunction-associated fatty liver disease; FIB-4, Fibrosis-4; HDL-C, high-density lipoprotein cholesterol; LDL-C, low-density lipoprotein cholesterol.

Table S3. Baseline characteristics of MAFLD participants with different subtypes

| Characteristic | MAFLD-diabetes (n=5826) | MAFLD-overweight/obese (n=99270) | MAFLD-lean  (n=1469) |
| --- | --- | --- | --- |
| Age, years | 59.70(6.98) | 56.46(8.01) | 57.86(7.89) |
| Male | 3780(64.9) | 61104(61.6) | 1201(81.8) |
| White ethnicity | 5104(87.6) | 91961(92.6) | 1299(88.4) |
| College or university degree | 1146(20.2) | 27052(27.7) | 430(29.7) |
| Townsend Deprivation Index | -1.06[-3.10,2.34] | -1.93[-3.53,0.97] | -1.80[-3.55,1.39] |
| Smoking status |  |  |  |
| Never | 2611(44.8) | 53891(54.3) | 707(48.1) |
| Former | 2562(44.0) | 35235(35.5) | 479(32.6) |
| Current | 653(11.2) | 10144(10.2) | 283(19.3) |
| Alcohol intake, g/day | 0.52[0.00,11.27] | 6.76[0.00,15.78] | 9.02[0.00,18.03] |
| Diet score |  |  |  |
| 0 | 1043(17.9) | 19263(19.4) | 313(21.3) |
| 1 | 2205(37.8) | 38875(39.2) | 595(40.5) |
| 2 | 1893(32.5) | 30505(30.7) | 424(28.9) |
| 3 | 1043(17.9) | 19263(19.4) | 313(21.3) |
| Physical activity,  MET-min/week | 1300.50[537.00,2819.25] | 1530.00[660.00,3182.00] | 1546.00[693.00,3279.00] |
| Insomnia |  |  |  |
| Never/rarely | 1132(19.4) | 25547(25.7) | 379(25.8) |
| Sometimes | 2472(42.4) | 46585(46.9) | 708(48.2) |
| Usually | 2222(38.1) | 27138(27.3) | 382(26.0) |
| BMI, kg/m2 | 33.56(5.36) | 31.58(4.32) | 24.13(0.79) |
| C-reactive protein | 4.10(5.65) | 3.60(4.77) | 3.78(5.41) |
| Triglycerides, mmol/L | 2.44(1.30) | 2.38(1.17) | 3.40(1.46) |
| Total cholesterol, mmol | 4.41(1.04) | 5.73(1.19) | 6.03(1.27) |
| HDL-C, mmol | 1.09(0.26) | 1.22(0.28) | 1.17(0.30) |
| LDL-C, mmol | 2.68(0.75) | 3.67(0.90) | 3.81(0.94) |
| Hypertension | 4322(74.2) | 35201(35.5) | 500(34.0) |
| History of Fracture | 518(8.9) | 8729(8.8) | 133(9.1) |

MAFLD, metabolic dysfunction-associated fatty liver disease; FIB-4, Fibrosis-4; HDL-C, high-density lipoprotein cholesterol; LDL-C, low-density lipoprotein cholesterol.

Table S4. Sensitivity analyses for the association of metabolic dysfunction-associated fatty liver disease with knee osteoarthritis

|  | Excluding cases that occurred within the first 2 years | |  | Accounting for competing risk of death | |  | Excluding participants with missing values | |
| --- | --- | --- | --- | --- | --- | --- | --- | --- |
|  | HR (95%CI) | P |  | HR (95%CI) | P |  | HR (95%CI) | P |
| Non-MAFLD | Reference |  |  | Reference |  |  | Reference |  |
| MAFLD | 1.19 (1.36-1.25) | <0.001 |  | 1.18 (1.30-1.24) | <0.001 |  | 1.16 (1.09-1.22) | <0.001 |
| **Fibrosis severity** |  |  |  |  |  |  |  |  |
| FIB-4 < 1.30 | 1.18 (1.12-1.24) | <0.001 |  | 1.16 (1.11-1.23) | <0.001 |  | 1.14 (1.07-1.22) | <0.001 |
| 1.30 ≤ FIB-4 < 2.67 | 1.20 (1.43-1.28) | <0.001 |  | 1.19 (1.13-1.25) | <0.001 |  | 1.17 (1.10-1.26) | <0.001 |
| FIB-4 ≥ 2.67 | 1.23 (0.99-1.46) | 0.056 |  | 1.27 (1.11-1.46) | <0.001 |  | 1.19 (0.99-1.41) | 0.059 |
| P for trend |  | <0.001 |  |  | <0.001 |  |  | <0.001 |
| **MAFLD subtypes** |  |  |  |  |  |  |  |  |
| MAFLD-diabetes | 1.07 (0.97-1.19) | 0.197 |  | 1.11 (1.01-1.23) | 0.023 |  | 1.09 (0.97-1.25) | 0.141 |
| MAFLD-overweight/obese | 1.20 (1.15-1.26) | <0.001 |  | 1.19 (1.14-1.25) | <0.001 |  | 1.16 (1.10-1.23) | <0.001 |
| MAFLD-lean | 1.16 (0.86-1.58) | 0.316 |  | 1.27 (0.97-1.68) | 0.082 |  | 1.48 (1.07-2.05) | 0.017 |

MAFLD, metabolic dysfunction-associated fatty liver disease; FIB-4, Fibrosis-4

Adjusted for age, sex, ethnicity, education level and Townsend deprivation index, physical activity, smoking status, daily ethanol intake, diet quality score, insomnia, body mass index, high-density lipoprotein cholesterol, low-density lipoprotein cholesterol, total cholesterol, triglyceride, and history of type 2 diabetes, hypertension, and fracture.
